# Obstructive sleep apnoea syndrome in children and anaesthesia

**DOI:** 10.4103/0019-5049.60491

**Published:** 2010

**Authors:** A Rudra, Manjushree Ray, S Sengupta, Asif Iqbal, G Maitra, S Chatterjee

**Affiliations:** Apollo Gleneagles Hospital, Kolkata, India; 1 N.R.S. Medical College, Kolkata, India; 2 Calcutta Medical College, Kolkata, India

**Keywords:** Anaesthesia, children, OSAS

## Abstract

Obstructive sleep apnoea syndrome (OSAS) is a common medical disorder among adults, which is increasingly being recognized in children too. It is a breathing disorder characterized by upper airway obstruction with or without intermittent complete obstruction that disrupts normal breathing during sleep. Anatomical and neuromuscular disorders are mainly responsible for this disorder. This disorder leads to a state of chronic hypoxemia, which has significant cardiac, pulmonary and central nervous system implications. Diagnosis of OSAS is based on thorough history and clinical examination along with appropriate sleep studies including polysomnography. The mainstay of treatment of paediatric OSAS is adenotonsillectomy. Good anaesthetic practice in Paediatric patients with OSAS revolves around good and ideal airway management. Early detection of airway obstruction, intense monitoring to warn of impending airway problems and appropriate and early intervention of airway compromise are good anaesthetic practices. Coexisting medical problems should be adequately addressed and safe analgesic techniques in the perioperative period go towards improving outcomes in patients with paediatric OSAS.

## INTRODUCTION

Obstructive sleep apnoea syndrome (OSAS) is a common medical problem in adults that is increasingly being recognized in children. It is a disorder of breathing during sleep characterized by prolonged upper airway obstruction and/or intermittent complete obstruction (obstructive apnoea), which disrupts normal ventilation during sleep and normal sleep patterns.[1]

OSAS occurs in children of all ages, but is most common in the two-eight years age group, when adenotonsillar hypertrophy is also common. The prevalence of OSAS in children is estimated to be approximately 2% and occurs equally among boys and girls.[2] This distribution contrasts with adults, in whom the prevalence is preponderant among men. Episodes of obstruction occur primarily during REM sleep, in contrast with obstructive apnoeas in adults, which occur in all sleep stages.

## TYPES OF SLEEP APNOEA

Types of sleep apnoea include:

- Central (absent gas flow, lack of respiratory effort)
- Obstructive (absent gas flow, upper airway obstruction and paradoxical movement of the rib cage and abdominal muscles)
- Mixed (due to both CNS defect and obstructive problems)

## PATHOPHYSIOLOGY

The aetiology and pathophysiology of obstructive sleep apnoea in children are multifactorial, with anatomic and neuromuscular abnormalities playing a major role in the disorder.[3] Others, however, consider neuromuscular factors of lesser importance because the vast majority of children with OSAS can be cured by correcting anatomic obstructions.[4] The narrowing of the airway lumen by hypertrophied lymphoid tissue, compliance and elasticity of the pharyngeal soft tissue, facial morphology, and the physiologic changes that occur in the pharyngeal dilators during sleep determine the severity of airway collapse.

Patients with dysmorphic constricted craniofacial development, such as those with Pierre Robin syndromes; and those with neuromuscular abnormalities as in cerebral palsy and anoxic encephalopathy, have a much higher incidence of severe OSAS.

Adenotonsillar hypertrophy plays a major role in the pathogenesis of OSAS in children. The volume of lymphoid tissue in the upper airway increases from about six months of age up to puberty, with maximum proliferation occurring in the preschool years, which coincides with the peak incidence of OSAS in children. Despite this narrowing of the upper airway by lymphoid tissue, most children do not develop OSAS. A normal child's airway is less likely to collapse in sleep than the adult airway.

This syndrome can cause significant cardiac, pulmonary, and central nervous system impairment due to chronic oxygen desaturation. Pulmonary vasoconstriction can increase pulmonary vascular resistance with resultant decrease in cardiac output due to cor pulmonale. Relief of the tonsillar/adenoidal obstruction can reverse many of these problems and prevent progression of others (pulmonary hypertension and cor pulmonale).

## CLINICAL FEATURES

Symptoms commonly described by parents of children with OSAS include nocturnal symptoms such as snoring, laboured breathing, paradoxical respiratory effort, observed apnoea, restlessness, sweating, unusual sleep positions (e.g., sleeping sitting up, hyperextension of the neck), and enuresis[5] The symptoms are almost always worse at the time of upper respiratory tract infection. Day time symptoms may include mouth breathing, poor school performance, excessive day time somnolence, morning headaches, fatigue, hyperactivity, aggression, and social withdrawal.

Unlike findings in adults, obesity is not a common factor in paediatric OSAS, although its role increases with the age of the child.[6] Other physical findings may include mouth breathing, retrognathia, or micrognathia. Tonsillar hypertrophy is a common physical finding in children with OSAS, although its absence neither excludes nor confirms the diagnosis.[7]

## DIAGNOSIS AND EVALUATION

The diagnosis of OSAS is based on a thorough history (frequent snoring and difficulty in breathing during sleep or obstructive apnoea observed by parents).

A recording of respiratory movement, expired carbon dioxide and pulse oximetry during sleep can be easily set up and these may help to confirm or refute the diagnosis. Nasal, laryngotracheal or other abnormalities must be sought by careful examination (e.g., by direct pharyngoscopy using a nasopharyngoscope) before assuming that OSA is due to enlarged tonsils and adenoid. Detailed polygraphic recording of respiration may be necessary to establish the diagnosis.

Polysomnography (PSG) remains the “gold standard” for the diagnosis of OSAS in adults as well as children. Normal PSG values for the various respiratory events have been reported by Marcus and Colleagues[8] [Table 1].

**Table 1:** Normal sleep study measurement in children

Measurement	Normal values
Sleep latency (min)	>10
Total sleep time (hours)	>5.5
% Rapid eye movement sleep	>15% TST
% Stage 3-4 non-REM sleep	>25% TST
Respiratory arousal index (No/hour TST)	<5
Periodic leg movements (No/hour TST)	<1
Apnoea index (No/hour TST)	<1
Hypopnoea index (nasal/oesophageal pressure catheter; No/hour TST)	<3
Respiratory disturbance index (Apnoea/hypopnoea index)	<1
Nadir oxygen saturation (%)	>92
Mean oxygen saturation (%)	>95
Desaturation index (>4% for 5 sec; No/hour TST)	<5
Highest CO_2_ (mm Hg)	52
CO_2_>45 mm Hg	<20% TST

TST: Total sleep time; REM : Rapid eye movement.

It was recommended to differentiate primary snoring, which does not require any form of treatment, from OSAS, and, if left untreated, can lead to cardiopulmonary dysfunction and functional impairment.[9] In general, studies have shown that history alone does not have sufficiently high diagnostic sensitivity or specificity to be the basis for recommending therapy.[10] Furthermore, polysomnography also may predict the success of treatment and postoperative complications[11] Although definitive diagnosis of OSAS is made by a positive polysomnogram, many children are not tested because the polysomnography laboratories specialising in children are few in number, and the tests are expensive and require an overnight stay.[12]

## AIRWAY IMPLICATIONS

Medical therapy of paediatric OSAS is not considered to be consistently effective. Systemic or topical steroids may shrink lymphoid tissue, but the long term effectiveness is not known, and a short course of systemic corticosteroids appears to be ineffective. Topical intranasal steroids appear to reduce the severity of OSAS.[13] Adenotonsillectomy remains the mainstay of treatment for paediatric OSAS.[14] Children with Down's syndrome deserve further comment because they frequently have severe obstructive sleep apnoea.[15] Although there are conflicting data on the usefulness of adenotonsillectomy in this group, it appears worthwhile if the tonsils or adenoids are obstructing the airway. If an adenotonsillectomy fails or is not considered appropriate therapy, uvulopalatopharyngoplasty may be effective.[16]

In infants and children in whom an adenotonsillectomy is contraindicated or in those patients who continue to be symptomatic after adenotonsillectomy, continuous positive airway pressure (CPAP) delivered through an appropriate nasal mask interface may be used to treat OSAS successfully.[17,18] Rarely, a tracheostomy may be necessary in very young patients, patients with craniofacial anomalies and neuromuscular syndromes, or patients who cannot tolerate CPAP or bilevel positive airway pressure (BiPAP) following the failure of adenotonsillectomy to resolve symptoms.

## PERIOPERATIVE MANAGEMENT OF THE CHILD WITH OBSTRUCTIVE SLEEP APNOEA

There is little specific evidence for or against the use of opiates and sedatives in the perioperative period in children with OSAS. To date, there are only anecdotal reports of respiratory depression in children in response to sedatives such as chloral hydrate[19] and respiratory depression in the postoperative period, including hypoxia.[20] Children with OSAS appear to have increased sensitivity to opioids. Because of the potential for such respiratory problems, all children with a history of OSAS should receive a comprehensive preanaesthesia evaluation and intensive intraoperative and postanaesthetic monitoring and care focused on the particular needs of the pathophysiology.

## SURGERY IN CHILDREN WITH OBSTRUCTIVE SLEEP APNOEA SYNDROME

Few studies provide data pertaining to complications of surgery in children undergoing adenotonsillectomy for upper airway obstruction. Supraglottic obstruction, breath holding, and desaturation on anaesthetic induction and emergence were the most common complications. Increased severity of OSAS, low weight, and young age are correlated with an increased rate of complications. Children with OSAS are at risk for respiratory complications after adenotonsillectomy, but these complications do not prolong the time to discharge.[20] Up to 20% of children with OSAS who undergo urgent adenotonsillectomy may experience such perioperative respiratory complications.[21] The authors of the articles define respiratory compromise in various ways but generally consider the need for supplemental oxygen as a main criterion. However, postoperative respiratory distress in children with OSAS often necessitates tracheal reintubation and mechanical ventilation or the use of non-invasive mechanical ventilation such as CPAP.[22,23]

Young age (younger than three years) and associated medical problems were found in most studies to define highest risk groups. A high preoperative respiratory disturbance index (apnoea/hypopnoea index) >40 or oxygen saturation nadir<70% or patients undergoing an additional uvulopalatopharyngoplasty also seems to be a risk factor for postoperative complications[23] Regardless of the type of surgical intervention, clinicians caring for children with OSAS during the perioperative period must consider risk factors for complications, proactively select anaesthetic and postoperative management techniques that minimize respiratory deterioration, employ vigilant monitoring practices, and proactively plan for handling acute respiratory failure.[22–24]

## PREANAESTHETIC EVALUATION

A review of the history, laboratory data, and physical examination results is essential for the optimal outcome of adenotonsillectomy.

During the preanaesthetic visit, the patency of oral and nasal air passages is carefully examined for the degree of tonsillar hypertrophy or inflammation and also the degree of nasal airway obstruction and adenoid hypertrophy. It is important to inspect the teeth routinely, because tonsillectomy is often performed on children losing their primary dentition. Because congenital malformations associated with difficult airways in children also may cause OSAS, therefore, special attention should be paid to the potential for difficult airway management in association with this population. When surgery for OSAS is performed on children with syndromes associated with difficult airways, alternative plans for establishing an airway should be arranged.[25,26]

In patients with severe OSAS, chest radiography, ECG, and possibly echocardiography to evaluate heart function may dictate a higher level of monitoring and postoperative care, depending on the results. If possible, the results of polysomnography also should be reviewed.

A detailed history of previous anaesthetic experiences, including the technique used for endotracheal intubation and response to opioid analgesics for postoperative pain control, are also important in anticipating perioperative issues.

## ANAESTHETIC MANAGEMENT

Children with OSAS are known to develop airway obstruction and episodes of apnoea in perioperative period. Therefore, all efforts should be made for early detection of such complications by intense monitoring using apnoea alarm, capnograph, pulse oximeter, precordial stethoscope, and electrocardiogram. If neuromuscular blockade is required, a neuromuscular monitor should also be used.

Patients should be premedicated with antireflux and antisialagogue drugs. Medical therapy of gastro oesophageal reflux consists of pharmacological interventions. The use of medication is aimed at blocking acid secretions using H_2_ blocker agents (e.g., ranitidine) and improving gastro oesophageal motility and gastric emptying (e.g., metoclopramide, bethanecol). Cisapride, a dopamine antagonist, is also used as a motility drug. Its mode of action is postulated to increase the release of acetylcholine from the myenteric plexus and to increase receptor sensitivity to acetyl choline. Furthermore, parasympatholytic drug (atropine 0.02 to 0.03 mg/kg) is indicated to prevent laryngospasm. Preoperative sedation with opioids or benzodiazepines or both should be used carefully with appropriate preoperative monitoring to prevent airway obstruction and hypoxemia.

Difficult airway may also be anticipated in children with OSAS. These children may have associated anatomical airway or craniofacial abnormalities. It is essential that the anaesthesiologist involved in the management of these children is skilled with advanced airway management techniques and equipped with equipment like fibre optic laryngoscope, laryngeal mask airways and alternate laryngoscope blades. Jaw thrust to prevent airway obstruction in these patients is more useful and superior to chin lift.

Inhalational induction technique is usually preferred in paediatric patients, but chances of airway obstruction are very high due to relaxation of genioglossus muscles.[27,28] Chances of airway obstruction significantly increase if patients receive fentanyl citrate prior to induction with inhalational agents.[29] The mouth must be kept open while the induction of anaesthesia until the gag reflex is abolished and oral airway is inserted. CPAP (10-15 cm H_2_O) helps to prevent pharyngeal airway collapse during induction of anaesthesia. Intubation should be done in a deeper plane of anaesthesia with or without intermediate acting non-depolarizing muscle relaxant. Cuffed endotracheal tube is preferred for tracheal intubation to reduce the chance of aspiration of blood and secretions and to reduce gas leaks around the tube. It is preferable to use a 0.5 to 1.0 mm ID smaller sized tube to accommodate the passage of the cuff through the sub glottis. There is evidence that high doses of dexamethasone (up to 1 mg/kg) reduce postoperative swelling, pain and decrease the incidence of PONV.

Extubation of an OSAS child is a challenge. Normally, extubation is done in a deeper plane of anaesthesia to prevent laryngospasm. However, this may be too risky in OSAS children. Therefore, current recommendation is to extubate OSAS child in a fully awake state with adequate airway muscle power. Extubation should be done in the operation theatre or postoperative care unit, where skilled personnel, medication and equipment for reintubation are readily available.

If the child does not breathe adequately after surgery, non-invasive ventilation can be instituted immediately after extubation.[12]

Regional block should be practiced, wherever possible, for perioperative analgesia. Systemic tramadol or paracetamol may be other alternatives to relieve postoperative pain.

Children with OSA who require sedation during magnetic resonance imaging of the upper airway, an effective sedation with limited effects on cardiovascular and ventilatory function could be achieved with a combination of ketamine (1 mg/kg) and dexmedetomidine (1 mc/kg). Sedation could be maintained without central apnoea by a continuous infusion of dexmedetomidine (1 mc/kg/hr).[30]

## POSTOPERATIVE CARE

At present, there are no guidelines and insufficient evidence to guide the decision–xmaking process of anaesthesiologists in the postoperative care of patients with obstructive sleep apnoea. However, patient's position, especially after extubation, seems to be important for the development of airway obstruction. Isono and associates, in a study of adult patients, reported that lateral position structurally improves maintenance of the passive pharyngeal airway in patients with obstructive sleep apnoea.[31] These findings are in concordance with the current practice of extubating and transporting children in the lateral position.

A major concern in the immediate postoperative period is the effect of residual anaesthesia, pain, sedative and analgesic medication, and oedema of the pharyngeal tissues on the complication rate in this category of patients. Therefore, children with severe OSAS or cardiovascular disease or those who are recovering from airway or craniofacial surgery should be monitored after surgery in the paediatric intensive care unit.

Children with OSAS are known to have a blunted ventilatory response to carbon dioxide, thus opioid analgesics, which will further depress respiratory drive and relax the pharyngeal dilator muscles, must be used judiciously during the postoperative period.[32] Optimal analgesia for children undergoing adenotonsillectomy for OSAS is controversial. Tramadol may represent a superior choice in this group with a potential to cause less postoperative sedation and respiratory depression[32] Dexamethasone 0.0625 – 1 mg/kg in combination with opioid provides better analgesia.[33]

To decrease nausea and vomiting the correct dose of dexamethasone may be 0.0625 mg/kg with no dose–escalation benefit.[34] Laryngospasm may be decreased by administering magnesium 15 mg/kg[35] or the “no touch” extubation technique.[36] Emergence delirium may be decreased with a single intravenous bolus dose of dexmedetomidine 0.5 *μ*g/kg given five minutes before the completion of surgery, thus providing a smoother transition to the postanaesthesia care unit.[37]

The anaesthetic management of OSAS should be directed toward assessing and managing the coexisting cardiac or pulmonary diseases; managing the airway, minimizing the amount of opiates used intraoperatively, and preventing and managing the possible postoperative complications. Preoperative sleep studies are necessary for a positive diagnosis and for decisions regarding postoperative monitoring.

It has been acknowledged that admission to an intensive care unit postoperatively is not recommended in all patients with OSAS (including patients with severe OSAS). However, all such patients should be closely monitored in the postanaesthesia care area for at least three hours after surgery. Depending upon the outcome in this period and the clinical judgement of the perioperative physician, the patient can be observed overnight in the high dependency area or in the general ward. Use of continuous positive airway pressure in the immediate postoperative period can reduce the incidence of respiratory complications and hence strongly recommended.
